# Ropivacaine Inhibits Lung Cancer Cell Malignancy Through Downregulation of Cellular Signaling Including HIF-1α *In Vitro*

**DOI:** 10.3389/fphar.2021.806954

**Published:** 2022-02-23

**Authors:** Junmei Shen, Lina Han, Yongxian Xue, Chao Li, Huiqun Jia, Kangsheng Zhu

**Affiliations:** ^1^ Department of Anesthesiology, The Forth Hospital of Hebei Medical University, Shijiazhuang, China; ^2^ Department of Blood Transfusion, The Forth Hospital of Hebei Medical University, Shijiazhuang, China; ^3^ Scientific Research Center, The Forth Hospital of Hebei Medical University, Shijiazhuang, China

**Keywords:** ropivacaine, HIF-1α, non-small-cell lung cancer (NSCLC), local anesthetics, proliferation, migration, invasion

## Abstract

**Background:** Ropivacaine is widely used to induce regional anesthesia during lung cancer surgery. Previous studies reported that amide-linked local anesthetics, e.g., ropivacaine, affected the biological behavior of lung adenocarcinoma cells, but the conclusion is controversial and warrants further study. This study set out to investigate the biological effects of ropivacaine on cultured lung cancer cells and underlying mechanisms.

**Methods:** Lung cancer cell lines (A549 and H1299) were cultured and then treated with or without ropivacaine (0.5, 1, and 2 mM) for 48 or 72 h. Their proliferation, migration, and invasion together with cell death and molecules including hypoxia inducible factor (HIF)-1α, VEGF, matrix metalloproteinase (MMP)-1, MMP-2, and MMP-9 expression associated with these changes were determined.

**Results:** Ropivacaine significantly inhibited proliferation and migration, invasion, and cell death in a concentration-dependent manner in both cell lines. Ropivacaine also promoted cell death and induced a concentration- and time-dependent cell arrest towards the G0/G1 phase. Expression of VEGF, MMP-1, MMP-2, MMP-9, and HIF-1α in both cell lines was also inhibited by ropivacaine in a concentration-related manner.

**Conclusion:** Our data indicated that ropivacaine inhibited lung cancer cell malignancy, which may be associated with downregulation of cell-survival-associated cellular molecules. The translational value of the current work is subjected to further study.

## Introduction

Lung cancer is one of the most common malignant cancers and causes the highest death among all cancers worldwide. Recent studies estimated that in 2020, the 5-year survival rate of lung cancer was only 19%, just behind pancreatic and liver cancer (Siegel et al., 2020). With the lung cancer screening strategy implemented recently, more and more early-stage lung cancer can be diagnosed, and patients can receive earlier surgical resection. However, lung cancer recurrence after surgery is still a clinical challenge. Perioperative risk factors including anesthetic use during surgery may contribute to cancer recurrence after surgery (Tavare et al., 2012; Wall et al., 2019), which may be due to anesthetics; in particular, inhalational anesthetics significantly modulated cell signaling changes, including hypoxia inducible factor (HIF)-1α (Huang et al., 2014; Unwith et al., 2015; Zhang W. et al., 2020). Conversely, it has been reported that local anesthetics (bupivacaine and levobupivacaine) have antitumor (colon, ovarian, and prostate cancer) properties (Xuan et al., 2015; Xuan et al., 2016; Li et al., 2019). Furthermore, clinical retrospective data also suggested that paravertebral anesthesia and analgesia for breast cancer surgery reduces the risk of recurrence or metastasis during the initial years of follow-up (Exadaktylos et al., 2006). Open prostatectomy surgery with general anesthesia, substituting epidural analgesia, was associated with substantially less risk of cancer recurrence (Biki et al., 2008). Patients who received paravertebral or high-pleural epidural anesthesia combined with sedation or light general anesthesia had a lower incidence of local or metastatic recurrence of breast cancer after surgery (Sessler et al., 2008; Snyder and Greenberg, 2010).

Ropivacaine is the most used amide-linked local anesthetic for regional anesthesia and acute pain, chronic pain, and cancer pain relief use (Yanagidate and Strichartz, 2007). Ropivacaine was reported to significantly inhibit the proliferation of gastric cancer cells, which was associated with reduction of the phosphorylation of EKR1/2 (Yang et al., 2018) and promoted liver cancer cell death *via* impaired mitochondrial function and caspase-3 activation (Wang et al., 2019). To understand the effect of ropivacaine on lung cancer malignancy and underlying mechanisms, the current study was set to investigate the effects of different concentrations of ropivacaine on proliferation, invasion, and metastasis of non-small-cell lung cancer cell lines and associated molecular changes.

## Materials and Methods

### Cell Culture

Human lung cancer cell lines (A549 and H1299), which are two of the common lung cancer phenotypes clinically, were purchased from the Cellular Biology Institute of the Shanghai Academy of Sciences (Shanghai, China) and cultured in RPMI-1640 medium (GIBCO, United States) supplemented with 10% bovine serum (Biological Industries, Beit HaEmek, Israel). The cells were grown in monolayer at 37°C in a humidified atmosphere supplemented with 5% CO_2_. Ropivacaine (AstraZeneca AB, Sweden) was dissolved in normal saline, with the pH adjusted to 7.4, and kept at −20°C. The cultured lung cancer H1299 and A549 cells at 90% confluence were treated with ropivacaine at 0.5, 1, and 2 mM. Cells treated with saline served as controls. Cobalt chloride (CoCl_2_) (Sigma-Aldrich, St Louis, MO, United States) at 100 μM was used to induce cellular hypoxia and increase HIF-1α expression in both A549 and H1299 cells treated with ropivacaine.

### Cell Proliferation Assessment

Approximately 5 × 10^3^ cells/well were placed in a 96-well plate and then treated with 0, 0.5, 1, and 2 mM ropivacaine for 48 or 72 h. Subsequently, MTS [3-(4,5-dimethylthiazol-2-yl)-5-(3-carboxymethoxyphenyl)-2-(4-sulfophenyl)-2*H*-tetrazolium] solution (Promega, Madison, WI, United States) (20 μl/well) was added to the cultures, which were incubated in the dark at 37°C for 2 h. The absorbance was measured at 492 nm with a microplate reader (Thermo Fisher Scientific Inc., MA, United States).

### Transwell Assay

The Boyden chambers (pore size 8 μm) (Collaborative Biomedical, Becton Dickinson Labware, Bedford, MA, United States) covered with or without 200 μg/ml Matrigel (Beyotime Biotechnology) were used to evaluate cell invasion or migration ability. A549 or H1299 cells (1 × 10^5^) were seeded in the upper chamber with 0.2 ml of RPMI 1640 medium without serum, while 0.6 ml medium with 10% FBS was added to the lower chamber. After incubation for 18 h, nonmigratory cells remaining above the membrane were removed with a cotton swab, and cells penetrating below the membrane are stained with crystal violet. Cells that penetrated the membrane were counted through a microscope in five randomly selected fields.

### Cell-Cycle Analysis and Apoptosis Analysis

The effect of ropivacaine in A549 and H1299 on cell-cycle progression was evaluated by flow cytometric analysis followed by propidium iodide (PI) staining. Cells were seeded in six-well plates and cultured for 24 h, and then the medium was replaced with no-serum medium for a further 24 h to synchronize the cell cycle at the G0/G1 phase; then the medium was replaced by ropivacaine (0, 0.5, 1, and 2 mM) for 48 or 72 h. After which, cells were stained with PI (MULTI SCIENCES, Hangzhou, China) according to the manufacturer’s instructions. The stained cells were incubated for 20 min at 37°C and then analyzed using a FACSCalibur flow cytometer (BD Biosciences, Franklin Lake, NJ, United States).

Approximately 5 × 10^5^ cells were gleaned and washed twice with phosphate-buffered saline (PBS) and then resuspended with 500 μl of 1× binding buffer. FITC annexin V and PI were added to the solution, which was then incubated in the dark for 5 min at room temperature. The cells were gently vortexed for flow cytometric analysis.

### Wound Healing Assay

Wound healing assay was performed to determine cell migration. The cells (5 × 10^5^) were seeded in six-well plates and cultured in the medium without bovine serum. When the cell confluence reached ≥80%, a 200 μl pipette was used to scratch a line on the monolayer gently. Then the medium was replaced by ropivacaine (0, 0.5, 1, and 2 mM) dissolved in culture medium. The micrographs of scratches were recorded randomly under an inverted microscope at 0, 24, and 48 h after being scratched.

### Western Blot Analysis

The proteins from the variously treated cells were extracted using RIPA buffer containing protease inhibitors. Lysates were centrifuged, and proteins were denatured through heating. The concentration of proteins was measured using the BCA assay (Beyotime, Nanjing, China). Total proteins (40 μg) were separated by 10% SDS-PAGE and then transferred to PVDF (polyvinylidene difluoride) membranes (Millipore, MA, United States). The membranes were blocked with 5% BSA for 2 h at room temperature and then were incubated overnight at 4°C with antibodies against matrix metalloproteinase (MMP)-2, MMP-9, MMP-1, VEGF, HIF-α, or GAPDH. Anti-rabbit IgG (Cell Signaling Technology, Boston, MA, United States) was visualized by Odyssey imaging (LI-COR, Lincoln, NE, United States). Antibodies against MMP-2, MMP-9, and MMP-1 were purchased from Cell Signaling Technology (CST, CA, United States). Antibodies against VEGF (polyclonal) and HIF-α were purchased from Bioss Biotechnology Co., Ltd. (Beijing, China), and GAPDH antibodies were obtained from Abcam (Cambridge, United Kingdom).

### Statistical Analysis

All data were expressed as mean ± standard deviation, and then comparisons to the mock treatments (controls) were made with nonparametric ANOVA first and followed with post-hoc Tukey’s test (SPSS version 19.0). A two-tailed *p*-value of less than 0.05 was considered statistically significant.

## Result

### Ropivacaine Suppresses Proliferation and Causes Apoptosis of Lung Cancer Cells

**FIGURE 1:**
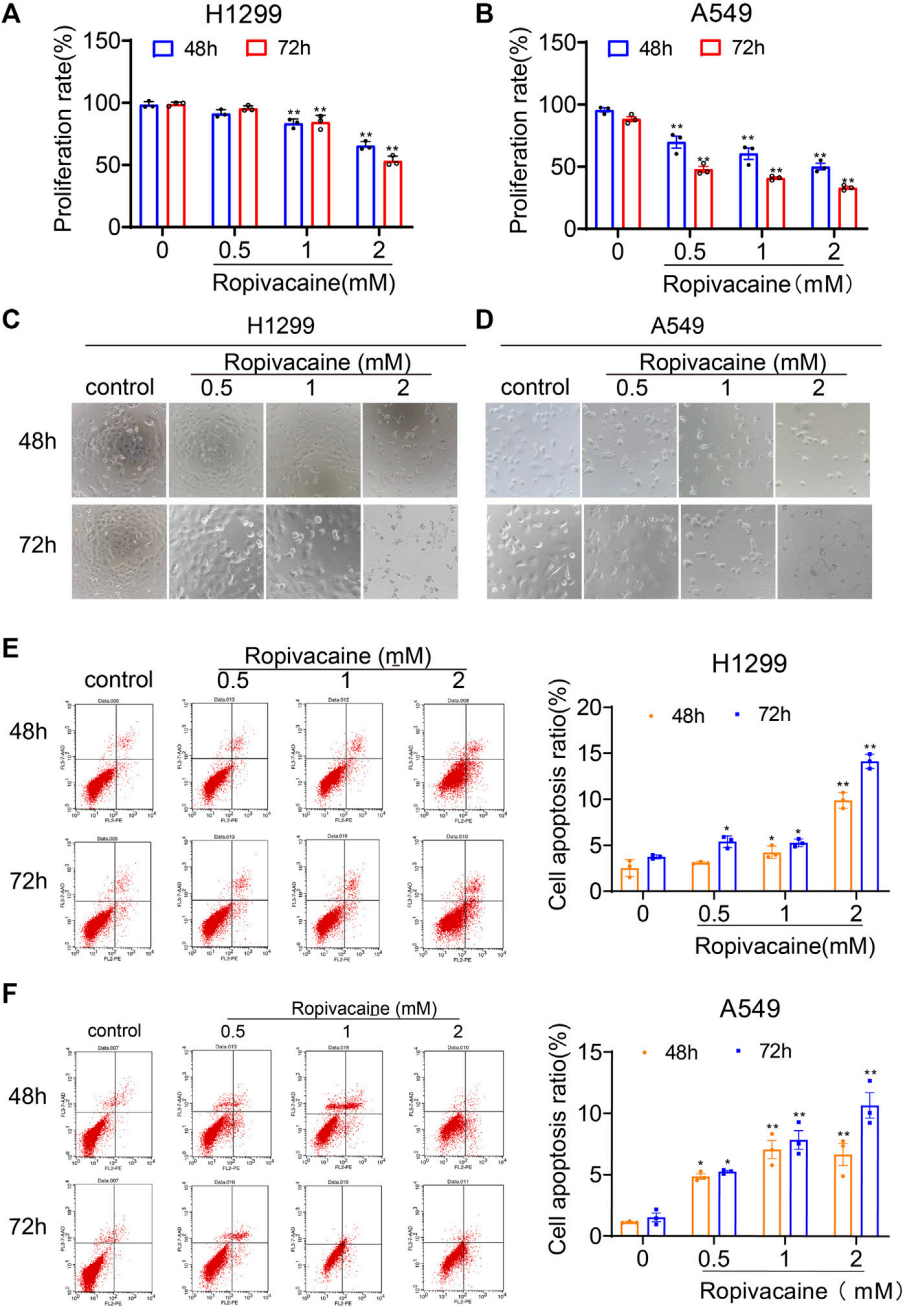
Ropivacaine inhibited proliferation and promoted apoptosis of lung cancer cells. H1299 **(A)** and A549 **(B)** cells treated with ropivacaine (0.5, 1, and 2 mM) for 48 or 72 h. Morphological changes of H1299 **(C)** and A549 **(D)** cells treated with ropivacaine for 48 or 72 h. Apoptotic death of lung cancer H1299 cells **(E)** and A549 cells **(F)** measured by flow cytometry analysis following a 48 or 72 h treatment. Independent experiments were repeated three times. Data are presented as the mean ± SD. *p < 0.05 and **p < 0.01 vs. the control group.

Ropivacaine inhibited the proliferation of lung cancer cells (Figure 1). For comparison, H1299 cells (Figure 1A) were less sensitive to ropivacaine exposure for either 48 h or 72 h than A549 cells (Figure 1B). The number of H1299 and A549 lung cancer cells was significantly decreased with an increase of ropivacaine concentration (Figures 1C,D). These changes, at least in part, were because ropivacaine caused the death of both types of cells via apoptosis (Figures 1E,F).

### Ropivacaine Induces Cell Cycle Arrest of Lung Cancer Cells

**FIGURE 2:**
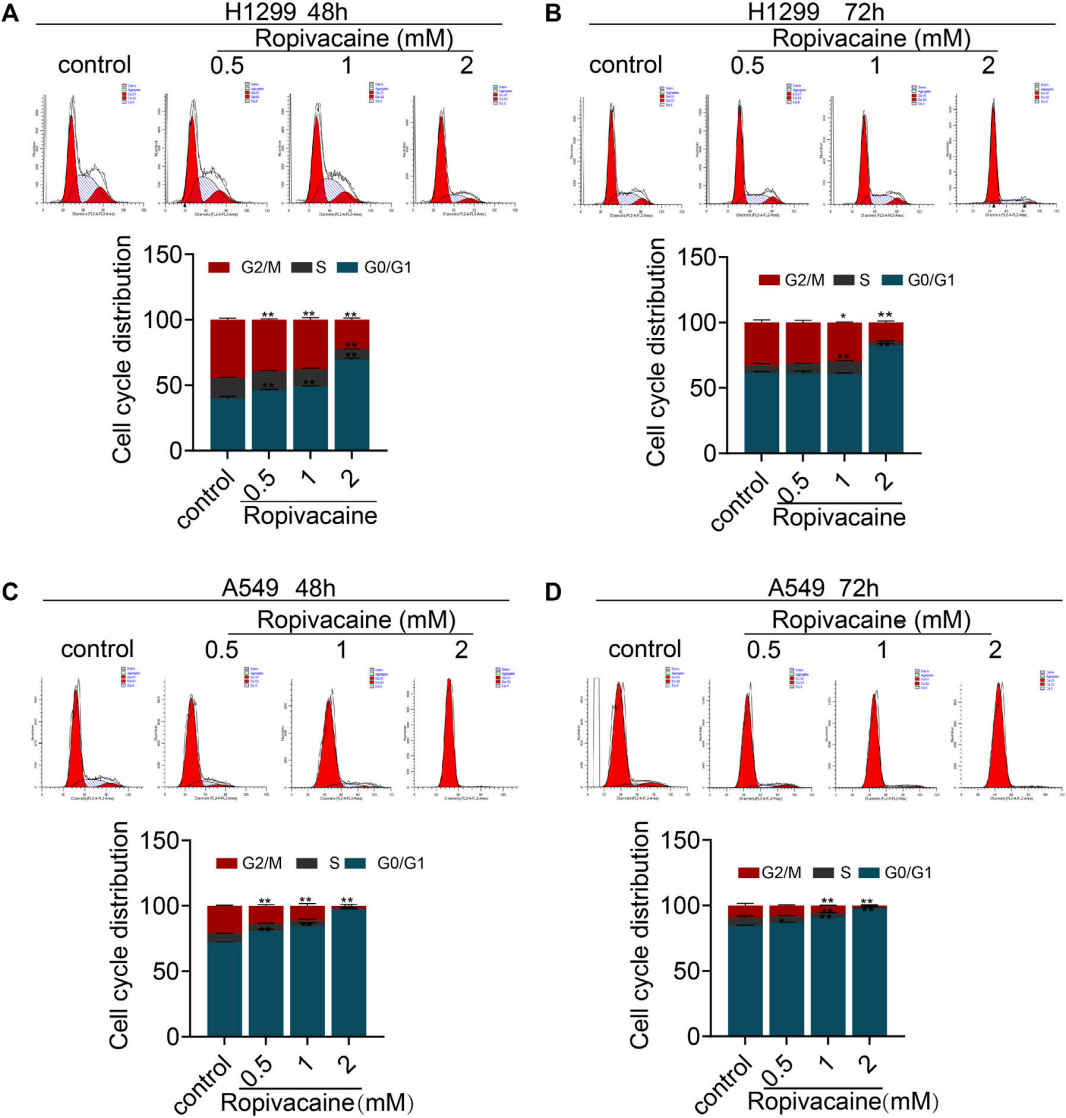
Cell cycle changes induced by ropivacaine. H1299 cells **(A**,**B)** and A549 cells **(C**,**D)** were treated with different concentrations of ropivacaine for 48 or 72 h. The cell cycle distribution was measured with PI staining and assessed with flow cytometry. Data are presented as the mean ± SD from three independent experiments. *p < 0.05 and **p < 0.01 vs. controls.

Considering that the ropivacaine inhibited the proliferation of H1299 and A549 cells, we further investigated its effect on cell cycle changes of lung cancer cells. Ropivacaine, in particular at 2 mM, induced cell cycle arrest in the G0/G1 phase, and this effect was more readily detectable in A549 than H1299 cells (Figures 2A–D).

### Ropivacaine Inhibited Lung Cancer Cell Migration and Invasion

**FIGURE 3:**
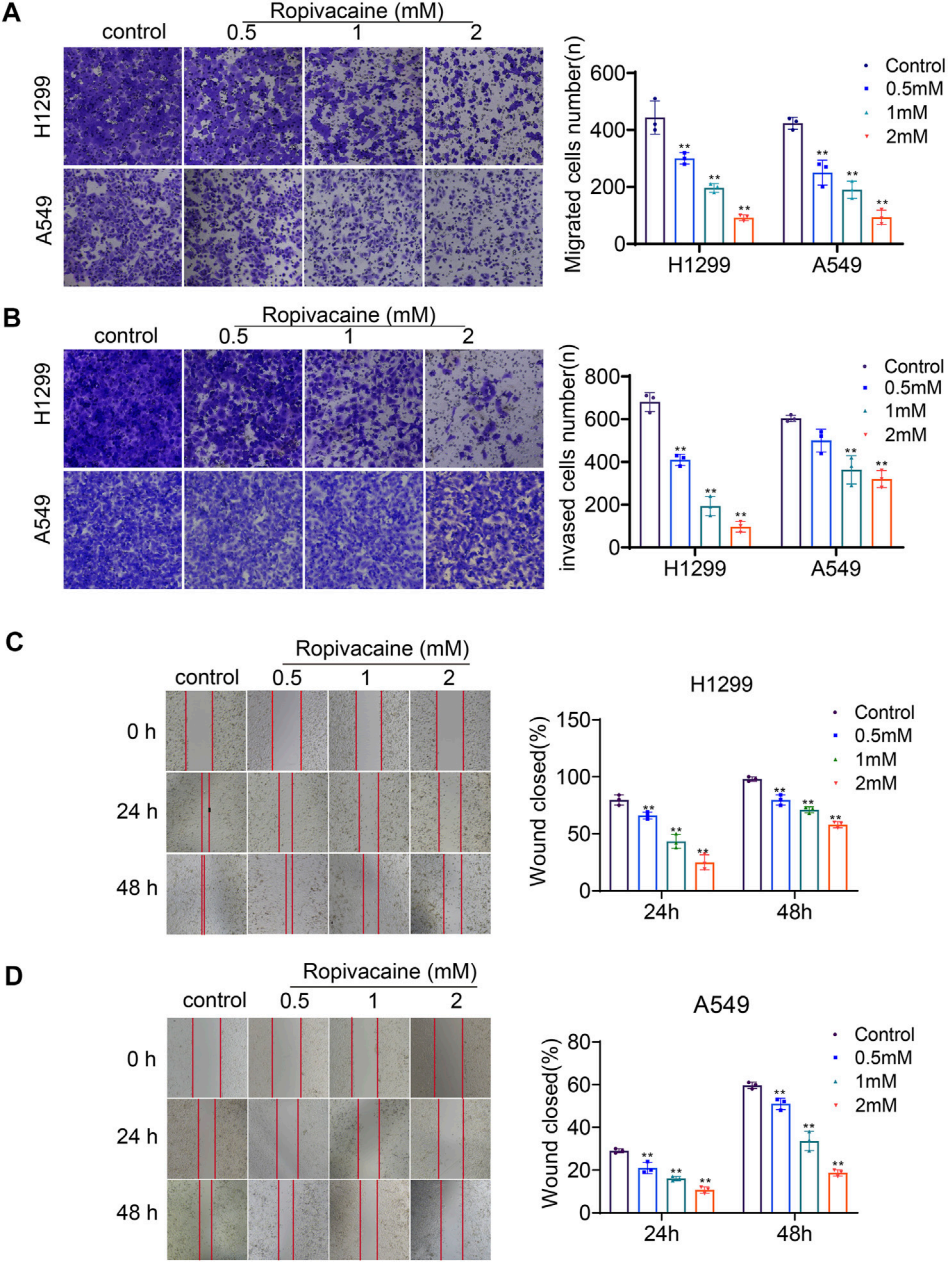
Ropivacaine treatment induced migration and invasion changes. **(A**,**B)** Effects of ropivacaine treatment on migration and invasiveness of H1299 and A549 cells were investigated using transwell and Matrigel assays. The number of cells that migrated or invaded was counted in five different fields. **(C**,**D)** Wound healing assays were performed to detect the migratory ability of H1299 and A549 cells, and the migratory ratio was determined by dividing the wound area by the total area. The data are expressed as mean ± SD from three independent experiments. *p < 0.05 and **p < 0.01 vs. controls.

The transwell assay and wound healing assay were applied to determine the effects of ropivacaine on the migration and invasion of H1299 and A549 cells. Cell migration assay data showed that the numbers of H1299 and A549 cells that migrated into transwell filters after treatment with ropivacaine at concentrations of 0.5, 1, and 2 mM were significantly reduced compared to those of the control group (*p* < 0.05) (Figure 3A). The invasion experiments showed that H1299 cells treated with 0.5, 1, or 2 mM ropivacaine had a significant decrease in the number of cells passing through Matrigel-coated membranes (*p* < 0.05) (Figure 3B). Similarly, the invasion ability of A549 cells treated with 1 or 2 mM ropivacaine was significantly decreased (*p* < 0.05) (Figure 3B). In the wound healing experiment, the wound healing process of H1299 and A549 cells was delayed significantly after treatment with 0.5, 1, or 2 mM ropivacaine at 24 and 48 h (*p* < 0.05) (Figures 3C,D).

### Ropivacaine Decreased VEGF, MMPs, and HIF-1α Expression of Lung Cancer Cells

**FIGURE 4:**
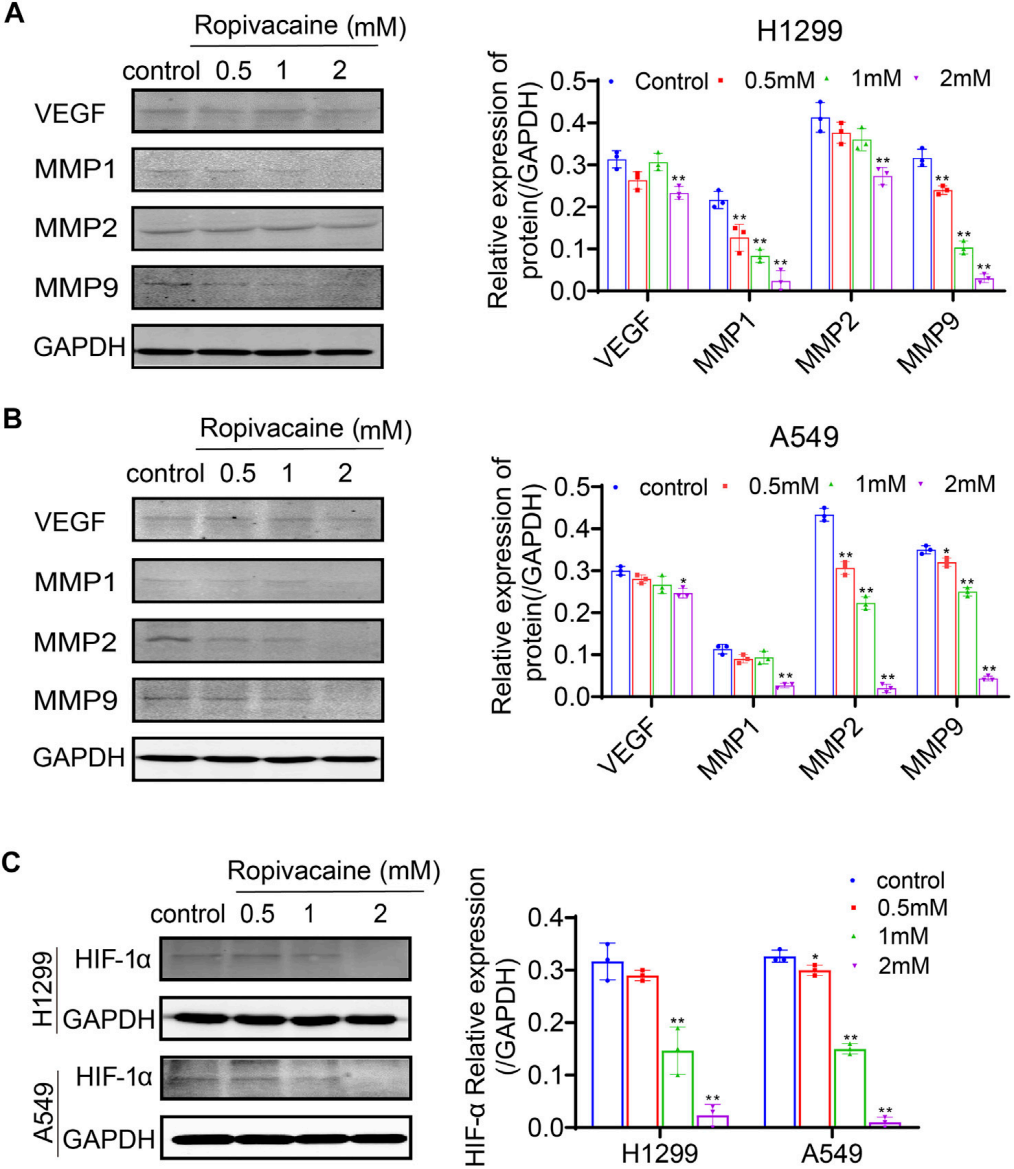
Ropivacaine treatment reduced expression of VEGF, MMPs, and HIF-1α in lung cancer cells. **(A)** H1299 cells and **(B)** A549 cells were treated with ropivacaine (0.5, 1, or 2 mM) for 48 h. The control group was treated with a no-FBS medium for 48 h. VEGF, MMP-1, MMP-2 and MMP-9 proteins in the cell lysates were assayed by western blot. GAPDH proteins were used as internal controls. **(C)** H1299 and A549 cells were treated with ropivacaine (0.5, 1, or 2 mM) and CoCl_2_ (100 μM) for 48 h. The control group was treated with a no-FBS medium and CoCl_2_ (100 μM) for 48 h. The protein expression of HIF-1α in H1299 cells and A549 cells was measured by western blot. The independent experiments were repeated three times. The data are expressed as the mean ± SD from three independent experiments. *p < 0.05, **p < 0.01 vs. controls.

VEGF and the MMP family were reported to be closely related to lung cancer malignancy and even metastasis (Merchant et al., 2017; Zhang et al., 2020a). To determine the mechanisms for why ropivacaine inhibited the invasion and metastasis of lung cancer cells, we detected by western blot the VEGF and MMP protein expression levels in H1299 and A549 cells after ropivacaine treatment. It was found that the protein expression level of VEGF in both H1299 and A549 cells treated with 2 mM ropivacaine was significantly lower than that in the controls (*p* < 0.05). The expression of MMP-1, MMP-2, and MMP-9 was also significantly decreased in a contraction-related manner after ropivacaine treatment in both H1299 and A549 cells (Figures 4A,B). VEGF and MMPs were downstream effectors of HIF-1α (Wang et al., 1995; Jiang et al., 1996). Therefore, we further detected the changes of HIF-1α in H1299 and A549 cells after ropivacaine treatment. To enhance HIF-α expression, cobalt chloride (CoCl_2_) was used to treat H1299 and A549 cells. Compared with that in the control group, the expression of HIF-1α in all treatment groups was significantly decreased, and the HIF-1α expression was decreased with the increase of ropivacaine concentration, especially at 1 and 2 mM (*p* < 0.05) (Figure 4C).

## Discussion

In the current *in vitro* study, we found that ropivacaine suppressed proliferation and invasion of lung cancer cells. Ropivacaine also promoted lung cancer cell death via apoptosis. Our data also demonstrated that ropivacaine significantly decreased migration ability of both lung cancer cell types. The cell cycle data indicated that ropivacaine arrested lung cancer H1299 and A549 cells staying in the G0/G1 phase. All these changes induced by ropivacaine may be associated with the decrease of MMPs, VEGF, and HIF-1α expression.

Tumor microenvironmental changes by any factors, e.g., inflammation induced by surgery, may promote cancer development and reoccurrence after surgery (Wall et al., 2019). Studies have shown that abnormal tumor microenvironments, such as hypoxia, pH value changes, and low glucose concentration, all affect the occurrence and development of tumors (Arneth, 2019). A hypoxic microenvironment is conducive to tumor growth and metastasis and plays a role in tumor initiation and progression (Liao et al., 2007). HIF-1α is a major subtype identified in the tumor microenvironment and has been found to be a key regulator of tumor growth (Pezzuto and Carico, 2018). In a normal-oxygen microenvironment, HIF-1α is hydroxylated by proline hydroxylase. Hydroxylated HIF-1α is suitable for binding to the tumor suppressor Von Hippel Lindau protein (VHL) in the cytoplasm that is being degraded by the protein body. Conversely, under hypoxia (1% O_2_ tension), proline hydroxylase is inactivated, and HIF-1α undergoes stabilization, nuclear translocation, and aggregation mechanisms and also evades decomposition via co-activator signals such as the C-terminal trans-activation domain (C-TAD) binding protein (CBP) (Kuschel et al., 2012). HIF-1α regulates a significant number of genes involved in many biological processes, including angiogenesis, glycolytic metabolism, and cell survival and invasion (Semenza, 2003). Overexpression of HIF-1α has been shown in many cancers. A previous study found that HIF-1α expression affected tumor proliferation and apoptosis in surgically resected lung cancer (Takasaki et al., 2016). HIF-1α is stable under low oxygen tension, so in our experiment, lung cancer cells were treated with cobalt chloride to obtain a hypoxic environment (Huang et al., 2003), and as a result, HIF-1α was upregulated in A549 and H1299 cells but decreased by ropivacaine in our study.

A significant association between MMP-9 and HIF-1α expression was reported in studies of lung cancers (Swinson et al., 2004; Chang et al., 2017). MMPs are zinc-dependent endopeptidases that participate in extracellular matrix degradation and play an important role in tumorigenesis, cell adhesion, and epithelial–mesenchymal transition. Among them, MMP-1, MMP-2, and MMP-9 are closely related to tumor invasion and metastasis (Nabeshima et al., 2002; Toth et al., 2012). It has been reported that ropivacaine can block tumor cell invasion and MMP-9 secretion (Piegeler et al., 2015). Our results showed that ropivacaine not only reduced the expression of MMP-9 but also inhibited the expression of MMP-2 and MMP-1 simultaneously. VEGF is one of the downstream effectors of HIF-1α (Jones et al., 2001; Pezzuto and Carico, 2018) and plays an important role in tumor development and even invasion and metastasis. These results suggested that ropivacaine effectively inhibited HIF-1α, VEGF, and MMP cellular signaling in human lung cancer cells and hence caused a decrease in malignant lung cancer cells. Similar to our results, previous studies also showed that ropivacaine inhibited the proliferation of breast cancer, cervical cancer, and thyroid papillary cancer cells; suppressed the invasion and metastasis of gastric cancer and thyroid papillary cells; and decreased the generation of tumor blood vessels (Zhang N. et al., 2020; Chen et al., 2020; Qin et al., 2020).

Our data may indicate that ropivacaine can change the lung cancer microenvironment; in particular, ropivacaine can potentially destroy new vascular formation and hence decrease the energy substrates supporting cancer cell development. Perhaps the most importance finding in our study is that ropivacaine directly suppressed lung cancer cell proliferation, migration, and invasion and promoted lung cancer cell death. All these effects may decrease the risk of lung cancer recurrence after surgery although this requires the direct application of ropivacaine to the cancer resection area, which is not a very common clinical practice during cancer surgery (Tavare et al., 2012; Wall et al., 2019). On the other hand, local anesthetics are often used for regional anesthesia, which can significantly block pain signal traveling through the pain pathway into the central nervous system, which causes surgical stress (Iwasaki et al., 2015; Xuan et al., 2015; Xuan et al., 2016). To this end, local anesthetics may have multi-beneficial effects for cancer patients.

Our work is not without limitations. Firstly, this study is a pure *in vitro* work, which is far from clinical settings. More clinical studies including animal studies are needed. Secondly, we found that ropivacaine was ineffective in both cancer cells in the µM concentration range found in our preliminary study. However, as stated above, local anesthetics are often given for local infiltration injection, and its concentration can reach to more than the mM range concentration. Hence, our data are clinically relevant although their translational value is subject to further study. Lastly, the causal relationship between the inhibitive effects of ropivacaine and molecular changes found in this study cannot be established and warrants further study.

In conclusion, our study suggested that ropivacaine inhibited the expression of HIF-1α in H1299 and A549 lung cancer cells, hence reducing the expression of its downstream effectors VEGF and MMPs and decreasing the ability of lung cancer invasion and metastasis potential *per se*.

## Data Availability

The raw data supporting the conclusion of this article will be made available by the authors, without undue reservation.
